# Induction of islet autoimmunity to defective ribosomal product of the insulin gene as neoantigen after anti-cancer immunotherapy leading to autoimmune diabetes

**DOI:** 10.3389/fimmu.2024.1384406

**Published:** 2024-03-26

**Authors:** Rene van Tienhoven, Diahann T. S. L. Jansen, Miso Park, John C. Williams, James Larkin, Sergio A. Quezada, Bart O. Roep

**Affiliations:** ^1^ Department of Cell and Chemical Biology, Leiden University Medical Center, Leiden, Netherlands; ^2^ Department of Internal Medicine, Leiden University Medical Center, Leiden, Netherlands; ^3^ Department of Cancer Biology and Molecular Medicine, Beckman Research Institute at City of Hope, Duarte, CA, United States; ^4^ Department of Medical Oncology, The Royal Marsden Hospital, London, United Kingdom; ^5^ Immune Regulation and Tumour Immunotherapy Lab, Cancer Immunology Unit, University College London (UCL) Cancer Institute, University College London, London, United Kingdom

**Keywords:** immune checkpoint blockade treatment, pembrolizumab, islet autoimmunity, neoantigen, defective ribosomal product, type 1 diabetes, insulinoma

## Abstract

**Introduction:**

The autoimmune response in type 1 diabetes (T1D), in which the beta cells expressing aberrant or modified proteins are killed, resembles an effective antitumor response. Defective ribosomal protein products in tumors are targets of the anti-tumor immune response that is unleashed by immune checkpoint inhibitor (ICI) treatment in cancer patients. We recently described a defective ribosomal product of the insulin gene (INS-DRiP) that is expressed in stressed beta cells and targeted by diabetogenic T cells. T1D patient-derived INS-DRiP specific T cells can kill beta cells and are present in the insulitic lesion. T cells reactive to INS-DRiP epitopes are part of the normal T cell repertoire and are believed to be kept in check by immune regulation without causing autoimmunity.

**Method:**

T cell autoreactivity was tested using a combinatorial HLA multimer technology measuring a range of epitopes of islet autoantigens and neoantigen INS-DRiP. INS-DRiP expression in human pancreas and insulinoma sections was tested by immunohistochemistry.

**Results:**

Here we report the induction of islet autoimmunity to INS-DRiP and diabetes after ICI treatment and successful tumor remission. Following ICI treatment, T cells of the cancer patient were primed against INS-DRiP among other diabetogenic antigens, while there was no sign of autoimmunity to this neoantigen before ICI treatment. Next, we demonstrated the expression of INS-DRiP as neoantigen in both pancreatic islets and insulinoma by staining with a monoclonal antibody to INS-DRiP.

**Discussion:**

These results bridge cancer and T1D as two sides of the same coin and point to neoantigen expression in normal islets and insulinoma that may serve as target of both islet autoimmunity and tumor-related autoimmunity.

## Introduction

Type 1 diabetes (T1D) immunopathology is characterized by insulitis in which islet-reactive CD8 T cells infiltrate the pancreatic islets of Langerhans and specifically target the insulin-producing beta cells (1, 2). The immune system is designed not to harm healthy self-tissue, and accumulating evidence suggests that stressed or dysfunctional beta cells provoke the immune system to attack by expressing aberrant or modified proteins (3–5). In this way, the autoimmune response in T1D resembles an effective antitumor response in which tumor (neo)antigens activate the immune system to respond and clear the tumor (4, 6).

In tumors, neoantigens derived from mutations, posttranslational modifications, and translational errors rather than native self-antigens, prime the immune system against the cancer. Some tumor neoantigens can result from the production of defective ribosomal products (DRiPs), which arise from alternative translation initiation, ribosomal frameshifting, and translation of normally untranslated regions (7). These DRiPs create a unique class of tumor-associated neoantigens that are highly immunogenic, as they do not contribute to thymic T-cell elimination and are currently explored as therapeutic targets to promote the anti-tumor immune response (8).

We discovered that stressed beta cells can produce a defective ribosomal product of the insulin gene (INS-DRiP) that serves as neoantigen and can provoke the immune system to attack. INS-DRiP is produced by alternative translation (in the +2 reading frame) of insulin mRNA during stress, leading to a non-stop protein (3). INS-DRiP-reactive T cells have been found in blood of T1D patients and in the insulitic lesion in the pancreas, and these T cells can specifically kill beta cells *in vitro (* 3, 9). Interestingly, islet-specific T cells have also been found in blood of non-diabetic individuals without causing autoimmunity (10–13), implying a peripheral regulation mechanism preventing such autoreactive T cells to cause harm.

PD1 expressed on CD8 T cells and PDL1 expressed by tumor cells and beta cells are immune checkpoint proteins that mediate peripheral tolerance (14, 15). The interaction of PD1 and PDL1 results in T-cell immune suppression, and tumor cells have adopted this strategy to escape immune detection (14). Immune checkpoint inhibitor (ICI) treatment aims to block the PD1–PDL1 interaction with therapeutic monoclonal antibodies, e.g., pembrolizumab or nivolumab that blocks PD1, thereby removing the brake from the immune system and unleashing immune reactivity by tumor-reactive T cells. This treatment has shown success to treat cancer (16), yet systemic ICI treatment can also activate other T cells that were suppressed by the PD1–PDL1 interaction, risking autoimmune adverse events including ICI-induced diabetes mellitus (“ICI-DM”) as side effect (17, 18).

Here, we report the induction of islet autoimmunity to INS-DRiP and activation of other islet-autoantigens upon ICI treatment with pembrolizumab, resulting in autoimmune diabetes. A monoclonal antibody was developed to detect INS-DRiP and revealed expression of INS-DRiP in insulinomas and confirmed our previous results in islets.

## Materials and methods

### Immune assays

Identification and quantification of HLA-A2-restricted islet-reactive CD8 T cells from cryopreserved peripheral blood mononuclear cells (PBMCs) were performed using the Diab-Q-kit, developed at Leiden University Medical Center (19, 20). The Diab-Q-kit uses a combinatorial encoding method whereby epitope specific CD8 T cells are identified by staining with a combination of two HLA class I multimers that have the same epitope, but different fluorochromes (Qdots, Invitrogen Thermo Fisher Scientific). This assay has been validated in international workshops. At least 50,000 events are required for a reasonable resolution, and our blood samples allowed between 664,000 and 1,333,000 events to be acquired. Costaining was performed with CD45RA (BioLegend) and CCR7 (BD Biosciences) to characterize the activation and differentiation status of islet autoreactive CD8 T cells. Data were acquired and analyzed on a BD LSRII using FACS Diva software.

### Immunohistochemistry

Pancreas coupes from organ donors and Whipple procedures of patients with insulinoma were used for immunohistochemistry. Paraffin-embedded human tissues were cut into 4-μm sections, deparaffinized in xylene, and rehydrated. Antigen retrieval was performed prior to staining with antibodies against insulin (Thermo Fisher Scientific, Cat. No. PA1-26938, RRID : AB_794668), glucagon (Cell Signaling Technology, Cat. No. 2760, RRID : AB_659831), somatostatin (Millipore, Cat. No. MAB354, RRID : AB_2255365), and a custom-made monoclonal antibody against INS-DRiP. Immunofluorescence was detected with a Zeiss LSM880 confocal microscope.

## Results

### Patient develops insulin-dependent diabetes after ICI treatment

Detailed patient information has been previously described (21). Briefly, a 54-year-old woman presented with a BRAF wild-type cutaneous melanoma that was resected in addition to a single positive axillary node. The patient relapsed 1 year after the initial presentation with subcutaneous metastases. Four doses of ipilimumab were given resulting in disease stabilization without significant side effects. After 8 months, the patient demonstrated disease progression and was treated with pembrolizumab three-weekly. While treatment was initially well tolerated, after three infusions, the patient was diagnosed with diabetic ketoacidosis, and insulin therapy was started. Further investigation revealed high serum titers of anti-GAD autoantibody (70.1 U/ml). Anti-insulin autoantibodies were in range (2.2 mg/l), and anti-islet cell autoantibodies were negative. Serum from before diabetes diagnosis was not available. HLA typing revealed high-risk HLA for developing T1D (HLA-DR4/DQ8 and HLA-A2).

### Islet autoimmunity post-ICI treatment

**Figure 1:**
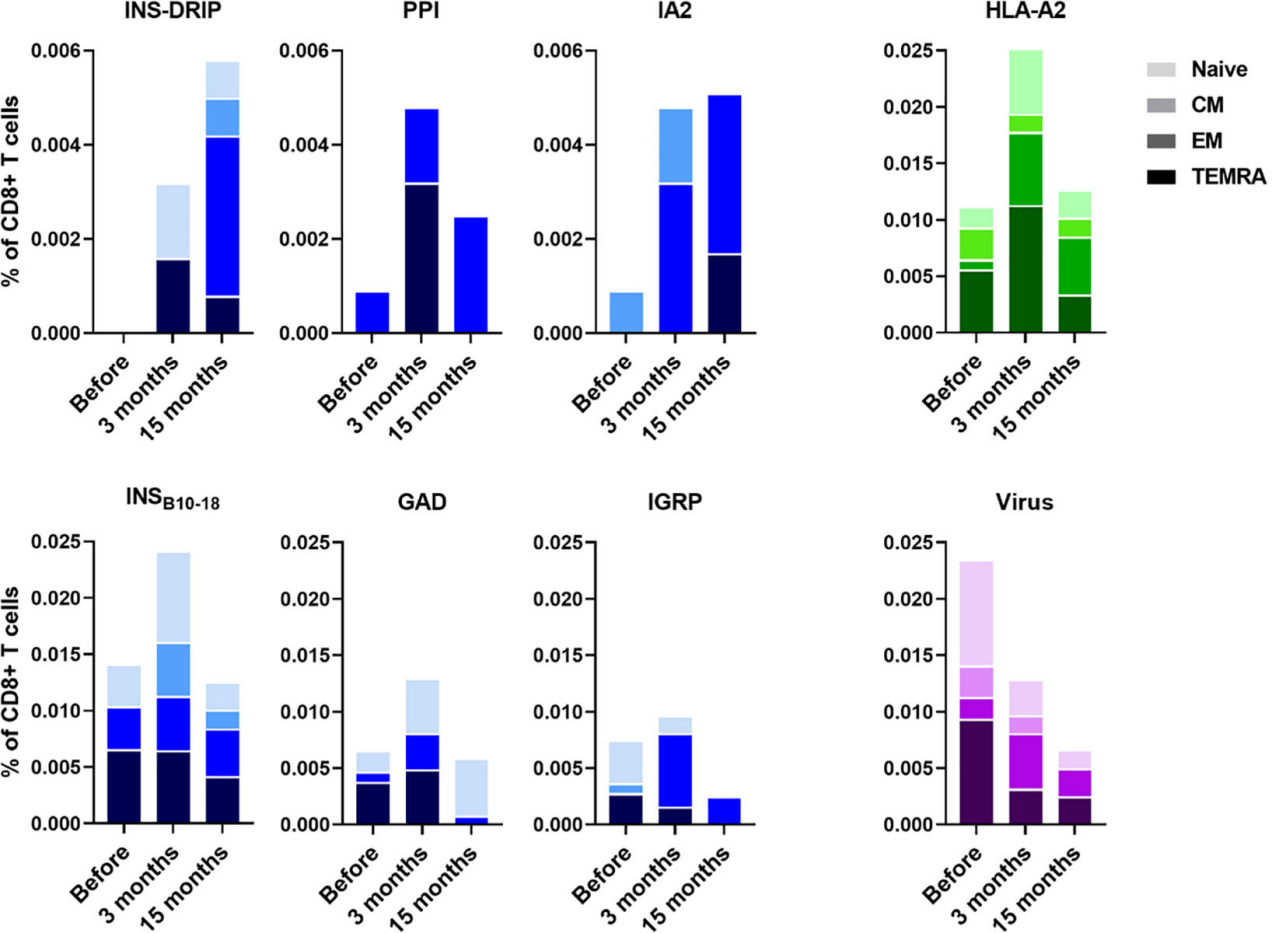
Immune responses to islet autoantigen before and after ICI treatment. PBMCs were collected before pembrolizumab treatment and 3 months and 15 months after. Islet-reactive CD8 T cells were quantified and phenotyped using the Diab-Q-kit in which two HLA-A2 multimers with two different fluorochromes are used for one epitope. Multimers contained peptides from the following islet antigens: PPI, INS_B10-18_, GAD, IA-2, and IGRP and the neoantigen INS-DRiP. Multimers against viral peptides (CMV, EBV, and measles) were used as control. HLA-A2 multimers containing a peptide derived from HLA-A2 was used as a measure for central tolerance. CD45RA and CCR7 were used to identify naive (CD45RA+ CCR7+), central memory (CD45RA− CCR7+; CM), effector memory (CD45RA− CCR7−; EM) and terminally differentiated effector memory cells (CD45RA+ CCR7−; TEMRA). Phenotypes are indicated by shades from dark to light: TEMRA, EM, CM, and naive.

PBMCs were isolated to monitor immune responses between ipilimumab and pembrolizumab treatment and 3 months and 15 months after. Diabetes was diagnosed 6 weeks after initiation of pembrolizumab treatment. CD8 T cells recognizing native islet autoantigens were present at baseline levels before ICI treatment, but increased 3 months after indicating that blockade of the PD1-PDL1 interaction indeed released autoreactive T cells from their peripheral immune regulation (**Figure 1**). CD8 T cells against neoantigen INS-DRiP were undetectable before ICI, but their frequencies increased at 3 months and rose even further at 15 months (**Figure 1**). T cells against IA-2 showed a similar pattern as those of INS-DRiP, whereas T cells to the preproinsulin signal peptide have peak levels at 3 months after ICI. The latter pattern was also seen for T cells against other islets autoantigens insulin B chain, GAD65 and IGRP. In addition to a difference in pattern based on the specificity, we observed a difference in phenotype and activation as well. CD8 T cells specific for INS-DRiP, PPI, IGRP, and IA-2 showed an increase in effector cells over time. At 15 months, CD8 T cells specific for PPI, IGRP, and IA-2 expressed an effector phenotype, while CD8 T cells to INS-DRiP, INS_B10-18_, and GAD had mixed phenotypes of naive and effector cells. These diverse patterns point to differential activation and expansion of islet autoreactive T cells.

All islet autoreactive CD8 T cells combined showed an increase in effector memory phenotype (19% before pembrolizumab therapy, 32% and 49% at 3 months and 15 months thereafter) and a decrease in TEMRA phenotype (44%, 30%, and 20%, respectively) over time after ICI treatment. The percentage of naive (31%, 27%, and 24%, respectively) and CM (6%, 11%, and 7%, respectively) islet autoreactive T cells was similar before and after treatment. The percentage of virus-specific CD8 T cells (CMV, EBV, and measles combined), to which no peripheral tolerance exists, showed opposite trends compared to autoreactive T cells, decreasing 3 months after ICI treatment and further decreasing after 15 months along with a decline of total CD8 T cells (18%, 15%, and 12% of PBMC, respectively; **Figure 1**). As reference of thymic immune function (central tolerance), we also tested an autologous peptide of HLA-A2 presented by HLA-A2. The percentage of HLA-A2 reactive CD8 T cells was increased at 3 months and back to baseline at 15 months (**Figure 1**), indicating incomplete thymic education at 3 months reflecting new T cells emerging shortly after pembrolizumab treatment. This seems most pronounced in case of the newly arising naive INS-DRiP specific T cells. Thus, ICI treatment induced INS-DRiP-specific CD8 T cells and increased and activated native islet autoantigen-specific CD8 T cells indicating that pembrolizumab indeed unleashed autoreactive T cells that were kept in check through peripheral immune regulation.

### INS-DRiP expression in pancreatic islets and insulinoma tissue

**Figure 2:**
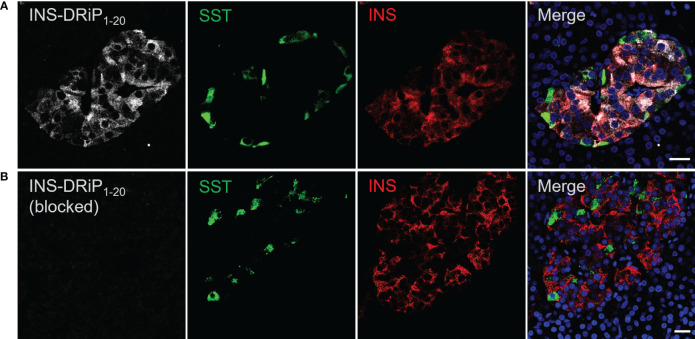
Monoclonal antibody against INS-DRiP-stained beta cells in human pancreas sections. **(A)** Immunohistochemistry of human pancreas sections with insulin (red), INS-DRiP (white), somatostatin (green), and Hoechst (blue). **(B)** INS-DRiP monoclonal antibody was blocked with immunization peptide prior to staining. Scale bars = 20 µm.

To assess expression of islet neoantigen in human islets, pancreas sections were stained with a monoclonal antibody against INS-DRiP in combination with insulin and somatostatin. INS-DRiP expression was determined in 12 non-diabetic cadaveric organ donors with consistent results. Representative images of one donor are shown (**Figure 2**). INS-DRiP colocalized with insulin in beta cells and was not detected in delta cells, confirming our previous results. INS-DRiP signal was not detected when the monoclonal antibody against INS-DRiP was blocked with immunization peptide, confirming the specificity of the antibody.

**Figure 3:**
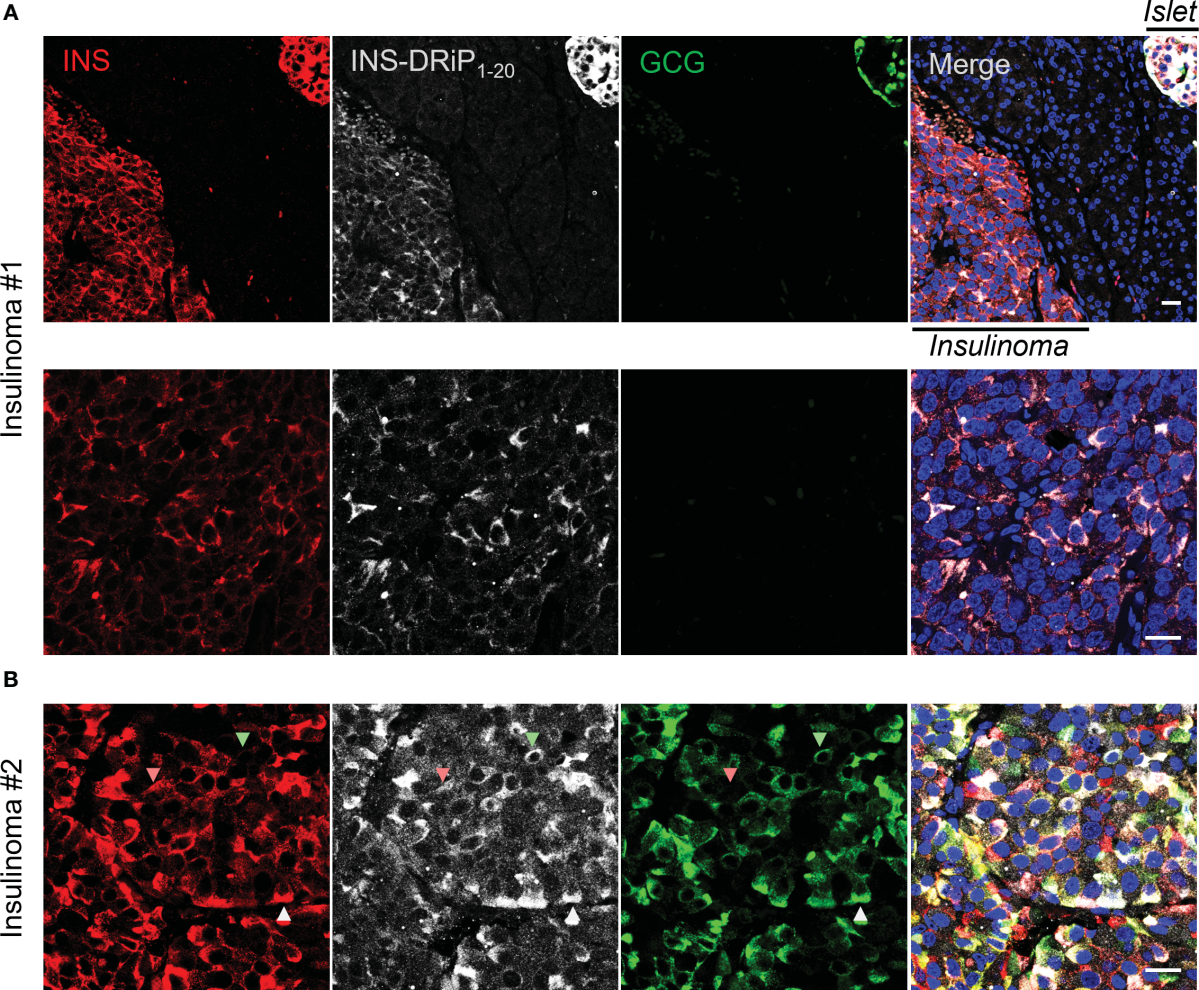
INS-DRiP is expressed in insulinoma tissue. Immunohistochemistry of Whipple procedure-derived human insulinoma sections with insulin (red), INS-DRiP (white), glucagon (green), and Hoechst (blue). **(A)** Islet–insulinoma interface (top panel) and insulinoma only (bottom panel) are shown for insulinoma 1. **(B)** Insulinoma 2 is shown. INS-DRiP colocalized with insulin+ cells (red arrow), glucagon+ cells (green arrow), and triple positive cells (white arrow). Scale bars=20 µm.

To bridge cancer and T1D, we tested whether cancerous beta cells from insulinoma patients express the same neoantigen as “diabetogenic” beta cells that are destroyed in T1D. Pancreas sections derived from Whipple procedures of two insulinoma patients were stained for INS-DRiP, insulin, and glucagon. INS-DRiP was detected in both insulinoma samples. In insulinoma 1, INS-DRiP colocalized with insulin in insulinoma tissue and beta cells of a neighboring islet, as shown on the islet–insulinoma interface (**Figure 3**). Insulinoma 2 showed both insulin and glucagon staining in the insulinoma tissue and INS-DRiP colocalized with insulin+ cells and glucagon+ cells, and triple positive cells. These results show that beta cells in islets and cancerous beta cells in insulinoma produce the same neoantigen INS-DRiP and indicate similarities between T1D and insulinoma.

## Discussion

We report the induction of autoimmunity to an islet neoantigen and activation of autoimmunity to native islet autoantigens in blood from a cancer patient treated with ICI, who developed diabetes as a side effect of successful cancer immunotherapy. We next demonstrate the presence of this neoantigen in a beta cell tumor (insulinoma), indicating the premise that this neoantigen may also serve as target in anti-tumor immunity, thus bridging autoimmunity and cancer therapy, be it in different individuals in this report.

The role of beta cells in their own demise has only recently been substantiated (4, 22). The generation of neoantigens in particular has caught attention, since such self-proteins are unlikely to contribute to thymic elimination and thus avoiding the induction of central immune tolerance, as reflected by the relatively high HLA binding affinity of neoantigenic peptides compared to native self-peptides (9). As a consequence, these proteins can act as vigorous provokers of immune responses that can lead to autoimmunity and the destruction of tissue. In case of cancer, this is desirable, as it leads to the elimination of the tumor. Indeed, neoantigens rather than native self-antigens are recognized by T cells upon inhibition of immune regulation by ICI (23), underscoring the efficacy of thymic education and induction of immune tolerance to self-proteins expressed in thymus, and the efficiency of peripheral immune regulation to control immune responses beyond the thymus.

In case of autoimmune diseases, the generation of neoantigens is an undesirable event that can lead to the activation of T cells destroying tissues, even if these are the source of vital hormones, as in case of T1D (3, 4). We have previously reported T-cell responses in T1D patients against neoantigens resulting from posttranslational modifications (24) (deamidation) and posttranscriptional modifications (3, 25) (alternative splicing and ribosomal infidelity). Importantly, such modifications are more likely to occur in the case of tissue distress, thereby exposing “danger signals” to the immune system, as would be desired in case of infection or cancer.

Although pancreatic tissues of non-diabetic donors were tested for INS-DRiP expression, the condition of the donors (trauma, ICU stay, medicine use, death, cold ischemia, among others) cannot be deemed as “normal”. Even in the case of a Whipple procedure, the metabolic status of the donor is abnormal given the presence of an endocrine tumor. The INS-DRiP expression in the islet adjacent to the insulinoma of this non-diabetic tissue appears even stronger than in the cancer cells and is in line with our proposition that INS-DRiP expression in both islets and tumor tissue can precede, and possibly even initiate, the development of islet autoimmunity and diabetes as a consequence of successful anti-tumor immunity.

The development of ICI-DM occurs in approximately 1% of treated cancer patients, and in particular in combination with induction of effective anti-tumor immunity, an in our case report. However, there is increasing awareness that the type of diabetes that developed as adverse event of ICI therapy is not identical with features most commonly seen in T1D (26). HLA-associated genetic predisposition to T1D may increase the risk for ICI-induced diabetes, as is the case in this report. Often, ICI-induced diabetes occurs in the absence of pre-existing islet autoantibodies and in patients with relatively normal HbA1c values in spite of excessively high blood glucose levels, pointing to an acute, *de novo* induction of diabetes, whereas T1D patients almost always show islet autoantibodies many years before diagnosis of T1D and early signs of dysglycemia before clinical disease manifestation. The frequent occurrence of diabetic ketoacidosis and undetectable or very low C-peptide levels in ICI-induced diabetes indicate a fast and fulminant decline of functional beta cell mass. The fact that we could detect autoreactive T cells to some islet autoantigens without diabetes even before ICI therapy is not unusual and points to the efficacy of peripheral immune regulation. Yet, our observations also imply that islet autoimmunity in the non-diabetic population is not necessarily benign and may cause harm if peripheral immune regulation is impaired (as in ICI therapy or infection). ICI-DM appears as discrete endotype of T1D. The cause of ICI-DM is subject to debate and may involve mechanisms other than autoimmunity in some ICI-DM cases. In our case, however, the data support the initial diagnosis autoimmune diabetes that was based on genetic predisposition and islet autoantibodies, adding T-cell autoimmunity to islet autoantigen as candidate pathogenic factor and underscoring islet autoimmunity as a component of ICI-DM. Yet, the most pronounced change in islet autoimmunity that we noted was against neoantigen INS-DRiP, which may imply that this could be a driving force in progression to diabetes. We recently demonstrated that the generation of INS-DRiP epitopes requires processing by the immunoproteasome in beta cells, which is accelerated by IFNγ, rather than IFNα (27). This may point to the need of concurrent local inflammation or insulitis for INS-DRiP specific T cells to kick in and destroy beta cells. We propose that ICI therapy adds to unleashing islet-autoreactive T cells that were kept in check by peripheral immune regulation and priming INS-DRiP-specific T cells. There are no data on such T-cell reactivity in insulinoma patients. We hope that our report will spark such studies, for which purpose our reagents (monoclonal antibody against DRiP and HLA-A2/DRiP tetramers) are available.

Our case report illustrates the notion that the immunopathogenesis of T1D resembles effective anti-tumor immunity. It also underscores that autoimmunity is the Achilles’ heel of ICI therapy in cancer. Closing the loop, we demonstrate that the neoantigen INS-DRiP to which autoimmunity was induced in this ICI-treated cancer patient was detectable in human insulinoma. We speculate that in the case of insulinoma as cancer, INS-DRiP may therefore also serve as neoantigen to which anti-cancer immunity may be unleashed by ICI therapy, at the risk of developing autoimmune diabetes as adverse event in those cases where the affected pancreas was not completely resected.

## Data availability statement

The raw data supporting the conclusions of this article will be made available by the authors, without undue reservation.

## Ethics statement

The requirement of ethical approval was waived according to NIH guidelines (https://grants.nih.gov/policy/humansubjects/hs-decision.htm) for the studies on humans. The studies were conducted in accordance with the local legislation and institutional requirements. Written informed consent for participation was not required from the participants or the participants’ legal guardians/next of kin in accordance with the national legislation and institutional requirements.

## Author contributions

Rv: Data curation, Formal analysis, Investigation, Methodology, Validation, Visualization, Writing – original draft, Writing – review & editing. DJ: Data curation, Formal analysis, Investigation, Methodology, Visualization, Writing – original draft, Writing – review & editing. MP: Methodology, Validation, Visualization, Writing – original draft, Writing – review & editing. JW: Methodology, Validation, Visualization, Writing – original draft, Writing – review & editing. JL: Data curation, Formal analysis, Investigation, Writing – original draft, Writing – review & editing. SQ: Conceptualization, Data curation, Formal analysis, Investigation, Methodology, Validation, Writing – original draft, Writing – review & editing. BR: Conceptualization, Data curation, Formal analysis, Funding acquisition, Investigation, Methodology, Project administration, Resources, Supervision, Validation, Visualization, Writing – original draft, Writing – review & editing.
